# A Narrative Review of Teledentistry in the AI Era: Scope, Opportunities, and Future Prospects in India

**DOI:** 10.7759/cureus.110008

**Published:** 2026-05-31

**Authors:** Arun K Simon, Sumeet Bhatt, Vaibhav P Thakkar, Mallikarjun Sajjanshetty, Sidra Bano, Thangadurai Maheswaran, Kaliyamurthy Sivaguru

**Affiliations:** 1 Public Health Dentistry, Sri Ramakrishna Dental College and Hospital, Coimbatore, IND; 2 Public Health Dentistry, Himachal Institute of Dental Sciences, Paonta Sahib, IND; 3 Public Health Dentistry, Mahatma Gandhi Mission (MGM) Dental College and Hospital, Navi Mumbai, IND; 4 Public Health Dentistry, Sri Balaji Dental College, Hyderabad, IND; 5 Oral Medicine and Radiology, Sri Ramakrishna Dental College and Hospital, Coimbatore, IND; 6 Oral and Maxillofacial Pathology, Adhiparasakthi Dental College and Hospital, Melmaruvathur, IND; 7 Prosthodontics, No. 1 Dental Unit, Assam Rifles, Shillong, IND

**Keywords:** artificial intelligence, digital dentistry, india, mobile health (mhealth), oral health, rural oral health, teledentistry

## Abstract

Teledentistry has evolved from pilot programs and COVID-19 adaptations into a clinically validated component of modern oral healthcare. In India, where access to dental care remains severely limited in rural populations, the convergence of teledentistry with artificial intelligence (AI) offers an urgent opportunity to reduce the burden of oral diseases. This narrative review examines the modalities and clinical evidence for teledentistry; awareness profiles and adoption barriers among Indian dental professionals and students; the diagnostic capabilities and readiness landscape for AI in dentistry; and the prospects, challenges, and implementation frameworks for AI-teledentistry integration in India. Evidence supports the diagnostic validity of synchronous and asynchronous teledental platforms for caries screening, oral lesion triage, and community surveillance. AI tools, including convolutional neural networks, large language models, and AI-generated educational content, enhance diagnostic accuracy, patient education, and resource optimization. However, formal training deficits, rural infrastructure gaps, limited oral health digital initiatives, and the absence of national regulatory frameworks remain critical barriers. A coordinated policy response that integrates AI into the dental curriculum and is supported by equity-centered implementation research is required.

## Introduction and background

Oral diseases remain the most prevalent preventable noncommunicable diseases globally, affecting an estimated 3.9 billion people and imposing a disproportionate burden on low- and middle-income countries, including India [1]. In India, a persistent paradox exists as follows: the country hosts over 460 million internet users and a rapidly expanding mobile subscriber base; however, dental care coverage in rural populations remains critically inadequate [1]. Teledentistry, defined as the application of telecommunication technologies to deliver dental care, consultation, education, and surveillance at a distance, offers a scalable mechanism to address this issue [2]. A scoping review of teledentistry in oral medicine spanning 1999 to 2021 identified India as the second-most productive contributor to the global teledentistry literature and found that 46% of the included publications addressed COVID-19-related applications, reflecting how the pandemic catalyzed its adoption worldwide [3]. Concurrently, artificial intelligence has emerged as a transformative force in dentistry as follows: a bibliometric analysis of 50 publications from 2020 to 2025 documented growing research clusters around machine learning, deep learning, generative AI, and clinical explainability [4], while a scoping review informing the 2025 Fédération Dentaire Internationale (FDI) policy statement mapped over 400 studies spanning AI, computer-aided design-manufacturing, digital imaging, and teledentistry as the principal pillars of digital dentistry [5].

Despite this promise, awareness surveys have revealed that most Indian dental professionals and students lack formal training in teledentistry or AI [6-9]. Post-pandemic surveys found that while 94.7% of Indian dental professionals were aware of teledentistry, only a small fraction had received structured education on its use [6]. Among dental students in South India, AI awareness was high at a surface level; however, knowledge of specific applications and software remained limited, particularly at the undergraduate level [7]. A translational research framework published by the International Association for Dental Research argues that digital inequities, driven by economic, regulatory, and workforce-related challenges, represent the central barrier to scaling e-oral health globally [10]. The Indian context amplifies both opportunities and challenges.

The literature for this review was identified through searches of PubMed using keywords related to teledentistry. Articles published in English between May 2017 and April 2026 were included in this review. Priority was given to recent reviews, clinical studies, guidelines, and landmark publications relevant to the scope of this narrative review. This narrative review explores the concept, modalities, and clinical applications of teledentistry; its awareness profile in India and the role of COVID-19 as a catalyst; the state of AI-based diagnostic tools and Indian readiness; and the prospects, barriers, and frameworks for converging AI with teledental platforms in India.

## Review

Concept, modalities, and clinical applications of teledentistry

Teledentistry is the use of telecommunication technologies to deliver or support dental care, consultations, education, and public health activities across geographic distances [2]. Telemedicine operates through the following two principal modalities: synchronous, involving real-time video or audio consultation between the clinician and patient, and asynchronous, involving store-and-forward transmission of clinical images, radiographs, and structured histories for later review [2]. A scoping review of teledentistry platforms classified the available communication tools into the following four categories: email, telephone, social network applications (including WhatsApp {Menlo Park, CA: Meta}, Zoom {San Jose, CA: Zoom Communications, Inc.}, Google Meet {Mountain View, CA: Google LLC}, and Viber {Tokyo, Japan: Rakuten Group, Inc.}), and purpose-built teledentistry systems, with store-and-forward delivery accounting for 30.71% of reported interactions, real-time consultations for 21.43%, and hybrid use for 35.71% [11]. Social network applications expanded markedly during the COVID-19 pandemic, and photographic recommendations for mobile-based oral examinations - addressing lighting, patient positioning, phone angle, and camera settings - were found to be critical determinants of image quality and diagnostic reliability [11]. A comprehensive narrative review published in 2025 confirmed that teledentistry has advanced from emergency pandemic use to a component of planned hybrid oral healthcare models, demonstrating gains in access, reduced travel burden, and high patient satisfaction, particularly among rural and institutionalized populations [2].

The clinical applications of AI span a wide range of dental fields. In community-based settings, teledentistry has been validated for caries screening in school-aged children. A study from rural India demonstrated that videographic assessment of dental caries yielded a sensitivity of 0.86, a specificity of 0.58, and an intraclass correlation coefficient of 0.56, with mean decayed, missing, and filled teeth index (DMFT) scores showing no statistically significant difference between videographic and visual-tactile methods (p=0.76), supporting the modality as a viable screening alternative for rural populations [12]. In oral medicine, remote screening for potentially malignant oral disorders was validated using WhatsApp-based photo messaging in rural Karnataka, where the sensitivity for lesion detection exceeded 98%, and kappa agreement values of 0.68 and 0.67 were recorded between two independent examiners [13]. The platform demonstrated substantial diagnostic agreement with clinical oral examination and was identified as a cost-effective adjunct in low-resource primary care settings [13]. A scoping review analyzing publications from 1999 to 2021 found that teleconsultation and telediagnosis were the most frequently employed modalities, with India ranking second in global publication output (11.86%), and identified seven categories of facilitators and six categories of barriers governing successful implementation [3].

Beyond diagnosis, a pilot study from remote Chile illustrated the use of satellite-enabled teledentistry for older adults, where 56% of participants had not visited a dentist in over five years, the mean DMFT was 24.2, and 84% of consultations involved emergency dental care [14]. The study highlighted that satellite connectivity can circumvent rural internet deficits and pointed toward the future integration of contextual AI to further strengthen these models [14]. Taken together, the evidence confirms that teledentistry functions best when embedded within hybrid care pathways that link remote screening and consultation with targeted in-person treatment and that standardized photographic protocols, reliable connectivity, and trained intermediaries are prerequisites for sustained, high-quality remote oral care [2,11]. A summary of the key clinical applications of teledentistry is provided in Table 1 [2,3,11-14].

**Table 1:** Key clinical applications of teledentistry: modality, setting, and evidence. AI: artificial intelligence; DMFT: decayed, missing, and filled teeth index; ICC: intraclass correlation coefficient

Application	Modality	Setting/population	Key finding
Dental caries screening	Videographic (store-and-forward)	Rural school children, India	Sensitivity 0.86; ICC 0.56; DMFT comparable (p=0.76)
Oral potentially malignant disorder screening	Photo messaging (WhatsApp)	Rural primary care, Karnataka, India	Sensitivity >98%; kappa 0.68-0.67
Emergency oral care in isolated populations	Satellite-enabled synchronous	Older adults, remote Chile	84% emergency consultations; DMFT: 24.2; future AI integration recommended
Oral medicine teleconsultation	Real-time/store-and-forward	Multicountry (India: second in publications)	Teleconsultation most used; 7 facilitators, 6 barrier categories identified
Orthodontics, pediatric dentistry	Hybrid (synchronous+monitoring)	Underserved and rural groups	High satisfaction; reduced travel; supports access equity
Image capture protocols	Store-and-forward (mobile camera)	Oral medicine settings	4 platform categories; photography standardization improves diagnostic reliability

Teledentistry awareness in India and the COVID-19 catalyst

Studies consistently report a satisfactory yet heterogeneous level of awareness of teledentistry among Indian dental professionals, with the COVID-19 pandemic emerging as the most powerful catalyst for attitudinal change. A survey of postgraduate dental students in Kanpur found that 74.4% had knowledge of teledentistry, 79.2% intended to practice it in the future, and the overall awareness-attitude score was 71.7% [9]. A broader post-pandemic survey of 431 Indian dental professionals recorded awareness of 94.7%, attributing this marked rise to the compelled adoption of remote platforms during COVID-19, when aerosol-generating dental procedures were restricted, and physical consultations were minimized [6]. COVID-19 effectively reframed teledentistry from a supplementary option to an operationally essential modality for the Indian dental workforce [6]. A recent cross-sectional survey of North Indian dental professionals found that 85.2% were aware of the term teledentistry, yet only 7.6% had received formal training; 88% expressed willingness to attend structured training if offered; and 79.7% indicated intent to use teledentistry in the future [15]. Among general dentists in Coimbatore, Tamil Nadu, 90% agreed that teledentistry represents a major future advancement, even though the majority acknowledged limited knowledge of its implementation, and 73% believed that it would meaningfully improve rural populations' access to dental specialists [8]. Recurrent barriers across all Indian surveys include inadequate infrastructure, limited formal training, concerns about diagnostic accuracy in complex cases, and uncertainty regarding applicability across all dental specialties [6,15]. Curriculum integration and continuing education programs are consistently recommended as the most impactful interventions for closing the knowledge-to-practice gap.

Diagnostic tools and Indian readiness for AI in dentistry

Artificial intelligence has rapidly emerged as a transformative force in dentistry, with applications spanning caries detection, periodontal assessment, determination of root canal morphology, prosthodontic design, and patient education [4,5]. A scoping review informing the 2025 FDI policy statement mapped 407 eligible articles and confirmed that AI constitutes one of the core domains of digital dentistry alongside computer-aided design (CAD) and computer-aided manufacturing (CAM) systems, computer-assisted surgery, and digital imaging, while underscoring that professional judgment and standardized, high-quality clinical evidence must anchor implementation [5]. A bibliometric review of AI and dentistry publications from 2020 to 2025 identified the largest thematic clusters around machine learning and deep learning, with an emerging trajectory toward generative and multimodal AI models, explainability, and fairness in clinical deployment, highlighting that responsible translation requires robust validation, transparency, and ethical oversight [4].

However, Indian readiness data reveal a significant gap between AI's promise and practitioner preparation. A cross-sectional survey of 208 dental students in South India found that 95.6% were broadly familiar with the term AI; however, only 25.8% of undergraduates were aware of specific AI applications in dentistry, compared with 90.9% of postgraduate students [7]. Critically, 80.6% of postgraduate students reported that AI was absent from their current curriculum, while 82.5% of undergraduates expressed interest in future AI learning, underscoring the urgent need for formal curriculum integration [7]. Among 338 Indian endodontists, fewer than half were aware of specific AI model architectures - 43.8% recognized artificial neural networks, and 35.2% were aware of deep learning models. Moreover, 91.1% had never operated AI software in clinical practice, and 89.3% explicitly requested formal training programs and workshops [16]. Despite these operational gaps, 82.5% of endodontists agreed that AI could enhance the success rate of endodontic treatment, affirming positive institutional attitudes [16]. Pediatric dentistry professionals in India similarly demonstrated knowledge deficits alongside positive attitudes toward AI, with cost, ethical concerns, and a lack of structured training identified as the principal barriers, and a significant association between awareness levels and years of clinical experience [17].

The potential of AI-enabled oral health education for underserved communities in India was demonstrated in an interventional study conducted in a tribal Adivasi ashram school in central India [18]. Children aged 10-15 years were exposed to a 3-min AI-generated animated video delivered daily for seven days in their local language, with the intervention producing statistically significant improvements in knowledge (mean difference 0.64), attitude (0.68), and practice (1.0), alongside a reduction in the gingival index from 1.30 to 0.55 and the plaque index from 1.41 to 0.51 (p<0.001) [18]. This represents the first reported use of a language-specific AI-generated video for oral hygiene promotion in a tribal school, illustrating that portable, culturally tailored digital tools can bridge health education disparities in remote Indian communities [18].

AI-teledentistry convergence, barriers, and future prospects for India

The convergence of AI and teledentistry creates a new paradigm for oral health delivery, which is particularly relevant to the epidemiological and infrastructural context of India. AI-enabled teledentistry supports remote diagnosis, treatment planning, monitoring, and follow-up by facilitating the transmission of high-quality intraoral images via smartphones, with AI tools pre-classifying images, flagging pathologies, and providing diagnostic support to remote clinicians [19]. AI also enables continuous monitoring of patients' oral health through real-time data from wearable and mobile devices, alerts clinicians to deterioration, and supports appointment scheduling and post-treatment follow-up across geographical barriers [19]. A systematic review of AI in oral health management concluded that AI can increase diagnostic accuracy, facilitate personalized treatments, optimize dental resource allocation, and provide early warnings for oral disease - outcomes that collectively amplify the reach and efficiency of teledental platforms [19]. The role of large language models within this ecosystem has been formally evaluated as follows: ChatGPT-4 (OpenAI: San Francisco, CA) achieved 90.5% accuracy on text-based oral and maxillofacial radiology questions and 90.0% accuracy on image-based questions, suggesting that large language models (LLMs) can support patient communication, clinical education, and teledental decision-making, albeit with acknowledged limitations in specialized or image-heavy tasks [20]. AI similarly supports disease surveillance, targeted prevention strategies, and equitable decision-making in public health-oriented teledental programs [21].

Despite this convergence, widespread translation into routine practice remains constrained by multilevel barriers. A translational research communication identified digital inequities - stemming from economic, regulatory, ethical, and workforce-related challenges - as the central impediment and proposed a three-pillar framework of technology and capabilities, workforce capacity, and real-world patient care to systematically advance e-oral health adoption [10]. A scoping review of dental patient-reported outcomes integrated into teledental consultations conducted across countries, including India, found that patient satisfaction, communication quality, and travel convenience were the most consistently measured outcome dimensions and called for standardized longitudinal evaluation frameworks [22]. For India, the mobile oral health (mOral health) context is particularly instructive, while the country hosts 460 million internet users and a rapidly growing mobile ecosystem, government-supported oral health digital initiatives remain limited to mCessation and the National Quitline, in stark contrast to a robust general health app portfolio that includes e-Raktkosh and mDiabetes [1]. The absence of a dedicated national oral health application, combined with the urban-rural digital divide, represents a structural gap that must be explicitly addressed in future policies [1].

Challenges identified in the literature include data inconsistencies across heterogeneous datasets, limited availability of annotated oral health data in Indian languages and populations, reliability concerns for AI models across diverse clinical environments, and algorithmic bias and fairness issues that affect equitable deployment [19]. Infrastructure deficits, particularly poor bandwidth and intermittent connectivity in rural areas, exacerbate these barriers. Ethical challenges, including patient data privacy, medicolegal accountability for AI-assisted diagnoses, and the absence of clear regulatory pathways for AI as a medical device, further complicate its adoption [10,21]. Addressing these impediments will require coordinated investment in digital infrastructure, AI, and teledentistry curriculum reform; national regulatory frameworks with reimbursement provisions; and equity-focused implementation research that prioritizes India's linguistic, geographic, and socioeconomic diversity. A summary of the barriers and strategic prospects for AI-based teledentistry integration in the Indian context is presented in Table 2 [1,10,15,16,19,21,22].

**Table 2:** Barriers and strategic prospects for AI-teledentistry integration in the Indian context. dPROM: dental patient-reported outcome measure; PG: postgraduate; UG: undergraduate

Domain	Current barrier	Strategic prospect
Workforce readiness	<10% formal training; 91% never used AI software	Mandatory AI modules in UG/PG dental curricula; hands-on workshops
Digital infrastructure	Poor rural bandwidth; intermittent connectivity	Satellite-enabled teledentistry; offline-capable AI tools
Oral health digital ecosystem	mCessation and Quitline only; no dedicated oral health app	National mOral Health platform integrating AI triage and education
Regulatory and ethical framework	No specific teledentistry/AI regulation in India	Evidence-based national policy; reimbursement provisions; data governance
Data quality and AI bias	Heterogeneous, limited, annotated Indian oral health datasets	Population-specific training datasets; fairness-aware AI algorithms
Patient-reported outcomes	Few standardized dPROM frameworks in teledental contexts	Longitudinal equity-focused trials; multilingual outcome instruments

## Conclusions

Teledentistry has matured from an emergency pandemic response into a clinically validated modality capable of bridging the vast oral health access gap in India, particularly in rural and underserved communities. Evidence consistently demonstrates that synchronous and asynchronous platforms achieve diagnostic accuracy that is broadly comparable to clinical examinations for caries screening, oral lesion triage, and community surveillance. The integration of AI deepens this potential; deep learning tools, large language models, and AI-generated educational content can augment the precision, scalability, and cultural relevance of remote oral care. India's expanding mobile infrastructure and status as a significant contributor to the global teledentistry research corpus provide a unique environment. However, significant structural barriers persist, including sparse formal training in both teledentistry and AI, inadequate rural connectivity, an underdeveloped oral health digital ecosystem, and fragmented regulatory guidelines. For clinicians, the most immediate, actionable step is to pursue structured training in validated teledentistry protocols and to advocate for the inclusion of AI in continuing dental education curricula. Broader impact will require national policy commitment, interdisciplinary collaboration between AI researchers and dental educators, and equity-centered implementation research that considers the linguistic, geographic, and socioeconomic diversity of India. Under these conditions, the AI-enabled teledentistry paradigm holds transformative potential for achieving oral health for all in India.
